# Supplementary material for: Detection of Equine Papillomaviruses and Gamma-Herpesviruses in Equine Squamous Cell Carcinoma
Source: Pathogens. 2023 Jan 23;12(2):179. doi: 10.3390/pathogens12020179 (PMC9958655; doi:10.3390/pathogens12020179)
Supplement: Supplementary file 1 [file pathogens-12-00179-s001.zip › pathogens-2101652-supplementary.pdf]

Supplementary File S1: Horse specifications, diagnosis, template description, PCR, and sequencing results

Table S1: Patient group:

Horse	Breed	Age	Color	Sex	Diagnosis	DNA from	EcPV	EHV
**SAMPLES FROM HORSES SUFFERING FROM SCC AND PRECURSOR LESIONS OF THE HEAD AND NECK (n = 33; from 22 horses)**								
ATH	Haflinger	22	sorrel	G	poorly cornified, poorly differentiated SCC sinus, oral	oral tumor tissue	NEG	NEG
BEL	Haflinger	22	sorrel	M	uncornified SCC of the maxillary sinus, ethmoid bone	tumor tissue	NEG	**2**
CHI	Trotter	23	bay	M	poorly differentiated maxillary SCC	tumor tissue	NEG	**5a**
DAN	Warmblood	22	chestnut	G	SCC nasal cavity & retropharyngeal LN metastases	tumor tissue	**2**	**5a**
FIL	Warmblood	26	chestnut	G	poorly differentiated, non-cornified SCC maxillary sinus	tumor sample	**5**	**5**
FYL	Icelandic horse	25	black	G	neoplastic lesions nasal septum, osteolysis	nasal swab	**2**	**POS**
GAY	Trotter	13	bay	G	SCC gingiva/palate (scirrhous compound, ulceration)	tumor tissue	NEG	NEG
GER	Trakehner	18	black	G	poorly differentiated SCC sinus, nasal cavity	tumor tissue	NEG	**2**
JON	Pony	25	chestnut	G	poorly differentiated SCC gingiva, palate & bone infiltration	tumor tissue	**2**	**2**
LEN	Warmblood	23	chestnut	M	SCC palatinal (210, 211), ventral nasal meatus	tumor tissue	NEG	NEG
MIL	Warmblood	22	chestnut	M	no written diagnosis. Papilloma or CIS of the sinus	tumor tissue	NEG	**2**
NAV	Trotter	12	chestnut	G	neoplasia oral cavity (palate, pharynx involved), neoplastic lesions prepuce & third eyelid right	eye tumor tissue	NEG	**POS**
						mandibular LN	**2**	NEG
						saliva	NEG	**5**
						nasal swab	NEG	**2**
						oral tumor tissue	NEG	NEG
NEP	Lusitano	21	grey	G	lingual SCC	tumor tissue	NEG	**5a**
						LN tissue	NEG	**POS**
OLG	Nonius	23	bay	M	nasopharyngeal neoplasia	tumor tissue	NEG	NEG
						genital swab	**2**	NEG
PRI	Shetland	26	black	G	cornified SCC sinus, nasal septum	tumor tissue	**2**	NEG
SAM	Haflinger-Warmblood	13	sorrel	G	pharyngeal SCC, metastases to retropharyngeal LNs	tumor tissue	**2**	**5**
						saliva	**2**	**5**
SPP[§]	Connemara	16	grey	M	SCC affecting right mandible and infiltrating lymphoid vessels, right LN metastases.	tumor tissue	**5**	NEG
						intact mucosa	NEG	**5a**

						PBMCs	NEG	**POS**
SHA	Pinto	17	skewbald	M	oral SCC affecting intermandibular space, tongue	tumor tissue	**2**	NEG
STA	Haflinger	7	sorrel	G	nasal SCC, suppurative foci, mandibular LN metastases	tumor tissue	NEG	**POS**
SUL	Pony	14	skewbald	G	severe nasopharyngeal tumor	tumor tissue	**2**	**5a**
						saliva	NEG	**5a**
						nasal swab	**2**	**5**
VAL	Icelandic horse	18	black	M	cornified SCC left dorsal nasal concha	tumor tissue	NEG	NEG
VUR	Icelandic horse	30	chestnut	G	2017: penile CIS; 2019: oral SCC rostral to epiglottis	oral tumor tissue	**2**	NEG
**SAMPLES FROM HORSES SUFFERING FROM (PERI-)OCULAR SCC AND PRECURSOR LESIONS (n = 36, collected from 24 horses)**								
ALI	Paint	25	overo	M	periocular CIS OD	tumor tissue	**2**	**2**
						PBMCs	NEG	NEG
ALO	Haflinger	22	sorrel	M	ocular SCC OS, precursor lesion OD	SCC OS	NEG	NEG
AMI	Criollo	12	paint	G	ocular SCC OS, ocular horny protrusion OD	SCC OS	NEG	NEG
						conjunctiva OD	NEG	**2**
						PBMCs	NEG	NEG
AND	Haflinger	13	sorrel	G	ocular SCC OU	oral SCC	NEG	NEG
						ocular SCC OD	NEG	NEG
ANE	Haflinger	6	sorrel	M	ocular SCC OD	tumor tissue	NEG	**POS**
BEL	Haflinger	14	sorrel	M	ocular SCC OD	conjunctiva OS	NEG	NEG
						SCC OD	NEG	NEG
BOD	Warmblood	19	chestnut	G	ocular SCC OD	tumor tissue	NEG	**5a**
						PBMCs	NEG	**2**
DAR	Paint	13	skewbald	M	ocular SCC OS	tumor tissue	NEG	NEG
DEX	Trakehner	16	piebald	G	ocular CIS OD	tumor tissue	NEG	**2**
FEE	Noriker	17	grey	M	ocular CIS/SCC OU	tumor tissue	NEG	NEG
HAN	Haflinger	11	sorrel	G	ocular precursor lesion OS	tumor tissue	NEG	NEG
JOH	Haflinger	11	sorrel	G	ocular SCC OD, precursor lesion OS	SCC OD	NEG	**POS**
KIR	Haflinger	19	sorrel	M	ocular SCC OD	tumor tissue	NEG	**5**
LAD	Haflinger	14	sorrel	M	ocular SCC OD	tumor tissue	NEG	NEG
MCH[§]	Connemara	15	grey	M		periocular lesion	**2**	**2**

					ocular SCC OS 2012: exenteration; 2014: poorly cornified periocular SCC OS, metastases parotis, oesophagus, trachea	parotid lesion	**2**	**2**
						tracheal LN	NEG	NEG
NAD	Warmblood	19	bay	G	ocular SCC OD	tumor tissue	NEG	**POS**
NAP	Warmblood	24	chestnut	G	ocular SCC OD	tumor tissue	NEG	**2**
NIN	Haflinger	4	sorrel	G	ocular SCC OD	tumor tissue	NEG	NEG
OCO	Welsh pony	17	grey	G	ocular SCC OD	tumor tissue	**4**	**POS**
						3rd eyelid	NEG	NEG
PAA	Warmblood	17	bay	G	ocular SCC OD	tumor tissue	NEG	**4** **5a**
						brush SCC	NEG	**5**
PUE	Haflinger	13	sorrel	M	ocular CIS/SCC OS 2012: keratectomy; 2015: infiltrative recurrent ocular SCC OS	CIS/SCC	NEG	NEG
						recurrent SCC	NEG	**5**
RUB	Haflinger	12	sorrel	M	ocular CIS/SCC OU	tumor tissue	NEG	**5**
STO	Haflinger	16	sorrel	G	ocular SCC OD, ocular precursor lesion OS	SCC OD	NEG	**5**
WIZ	Haflinger	5	sorrel	G	ocular SCC OD, papilloma OS	ocular SCC OD	NEG	**POS**
						ocular swab OS	NEG	**POS**
**SAMPLES FROM MALE HORSES SUFFERING FROM GENITAL SCC AND PRECURSOR LESIONS (n = 53, collected from 36 geldings)**								
ARO§	Hanoverian	18	bay	G	penile SCC	tumor tissue	**2**	NEG
ALV	Icelandic horse	24	chestnut	G	penile SCC	tumor tissue	**2**	NEG
						smegma	**2**	**3**
BAK	Icelandic horse	27	chestnut	G	penile SCC	tumor tissue	**2**	NEG
BIK	Icelandic horse	30	chestnut	G	penile SCC	tumor tissue	**2**	**2**
BLT	Icelandic horse	25	black	G	penile SCC	tumor swab	**2**	NEG
						ocular swab right	NEG	NEG
						ocular swab left	NEG	**5**
						smegma	**2**	NEG
BLZ	Icelandic horse	20+	black	G	penile CIS	tumor tissue	**2**	**5**
BLÁ	Icelandic horse	23	chestnut	G	penile SCC	tumor tissue	**2**	NEG
BOG§	Nonius	28	black	G	penile SCC	tumor tissue	**2**	NEG
						intact skin	**2**	NEG
BRY§	Icelandic horse	24	black	G	penile SCC	tumor tissue	**2**	**2**
DIS	Warmblood	16	bay	G	penile SCC	tumor tissue	**2**	NEG -

						smegma	**2**	**POS**
EVN	Icelandic horse	28	bay	G	penile SCC, massive tissue destruction	tumor tissue	**2**	**2**
ERC	Warmblood	15	bay	G	penile SCC (apical) and papilloma	SCC tissue	**2**	NEG
						papilloma tissue	**2**	NEG
FIL	Pony	18	?	G	penile papilloma	tumor tissue	**2**	NEG
FRI§	Warmblood	21	chestnut	G	penile papilloma	tumor tissue	**2**	**5a**
						ocular swab	NEG	NEG
HJÁ	Icelandic horse	14	dun	G	penile SCC	tumor swab	**2**	NEG
						tumor tissue	**2**	NEG
JAC	Haflinger	17	sorrel	G	penile SCC (apical)	tumor tissue	**2**	NEG
						cells ex tumor	**2**	NEG
JIM	Arabian	13	grey	G	penile CIS	tumor cytobrush	**2**	NEG
KAL	Arabian x PRE	21	grey	G	penile SCC, plaques	plaque tissue	**2**	NEG
KRO	Icelandic horse	28	?	G	penile SCC	smegma	**2**	NEG
LEI	Icelandic horse	5	sorrel	G	penile plaques	tumor tissue	**2**	**2**
MAX	Haflinger	21	sorrel	G	penile SCC	cells ex tumor	**2**	NEG
MB1	Quarter horse	19	chestnut	G	penile SCC	smegma	**2**	NEG
						tumor tissue	**2**	**5**
MB2	Welsh pony	29	chestnut	G	penile SCC	tumor tissue	**2**	**5a**
						smegma	**2**	**5a**
MB3	Irish cob	19	piebald	G	penile SCC	tumor tissue	**2**	NEG
MEX	Cross breed	20	bay	G	penile SCC	tumor tissue	**2**	**2**
MEL	Cob	15	piebald	G	penile SCC	tumor tissue	**2**	**3**
MRC	Irish hunter	18	?	G	penile SCC	tumor tissue	**2**	NEG
NAT	Icelandic horse	29	chestnut	G	penile SCC	tumor tissue	**2**	NEG
SAH	Trakehner	26	bay	G	penile SCC	tumor tissue	**2**	NEG
SHN	Criollo	18	skewbald	G	penile SCC	tumor tissue	**2**	**5a**
						tumor tissue	**2**	**5a**
SPO	Paint	20	chestnut	G	penile CIS	tumor tissue	**2**	**2**
STA	Haflinger	15	sorrel	G	penile SCC	tumor tissue	**2**	NEG
						PBMCs	NEG	**2**

STE	Icelandic horse	24	grey	G	penile SCC	tumor tissue	**2**	NEG
TAL§	Warmblood	23	bay	G	penile SCC	tumor tissue	**2**	**5a**
TVI	Icelandic horse	27	bay	G	penile SCC	tumor tissue	**2**	NEG
						smegma	**2**	**5**
						PBMCs	NEG	**2**
						tumor swab	**2**	**5**
WIN	Pony	25	skewbald	G	penile SCC	tumor tissue	**2**	**5**
**SAMPLES FROM FEMALE HORSES SUFFERING FROM GENITAL SCC AND PRECURSOR LESIONS (n = 7, collected from 7 mares)**								
ALC	Haflinger	21	sorrel	M	clitoral SCC	tumor tissue	**2**	NEG
ELM	Shetland pony	22	grey	M	vulvar CIS	tumor cytobrush	**2**	NEG
EXV	no information	?	?	M	vulvar SCC	tumor tissue	**2**	**5a**
GJE	Warmblood	20	skewbald	M	vulvar, poorly differentiated SCC	tumor tissue	**2**	NEG
JEA§	Haflinger	29	sorrel	M	vulvar SCC	tumor tissue	**2**	NEG
MB4	Warmblood	20	?	M	clitoral SCC	tumor tissue	**2**	NEG
SVP	Icelandic horse	16	chestnut	M	vulvar SCC	tumor tissue	**2**	NEG

EcPV: equine papillomavirus; 2 = EcPV2, 3 = EcPV3; 4 = EcPV4; 5 = EcPV5; EHV: equid herpesvirus; 2 = EHV2; 3 = EHV3; 5 = EHV5; 5a = AsHV5; NEG: negative; POS: Herpesvirus PCR positive, but herpesvirus type not identified; M: mare; G: gelding; LN: lymph node; SCC: squamous cell carcinoma; CIS: carcinoma in situ; OD: oculus dexter; OS: oculus sinister; OU: Oculus uterque; PBMC: peripheral blood mononuclear cells; in bold: positive detection results.

§ Tumour samples from these horses also tested positive for EcPV2 E6 (and E1) transcripts as reported previously (Sykora, S., L. Samek, K. Schönthaler, F. Palm, G. Borzacchiello, C. Aurich, and S. Brandt. 2012. EcPV-2 is transcriptionally active in equine SCC but only rarely detectable in swabs and semen from healthy horses. Vet Microbiol 158(1-2):194-198.)

Table S2: Control group

Horse	Breed	Age	Color	Sex	Reason for Vet consult	DNA from	EcPV	EHV
**SALIVAL AND PERIODONTAL FLUID SAMPLES FROM TUMOR-FREE HORSES (n = 31, from 31 horses)**								
ARD	Icelandic horse	15	bay	M	dental consult	periodontal fluid	NEG	NEG
ARN	Haflinger	12	sorrel	G	dental consult	periodontal fluid	NEG	**POS**
ATT	Warmblood	21	chestnut	S	dental consult	saliva	NEG	NEG
BGB	Warmblood	17	bay	G	dental consult	saliva	NEG	NEG
BILL	Warmblood	19	bay	G	healthy	saliva	NEG	**5a**
BIV	Fjord pony	17	buckskin	S	dental consult	saliva	NEG	NEG
BLT	Icelandic horse	22	black	G	dental consult	saliva	NEG	NEG
BOL	Warmblood	15	bay	G	dental consult	saliva	NEG	NEG
CHB	Warmblood	22	bay	G	dental consult	saliva	NEG	NEG
DAD	Warmblood	7	bay	G	spinal ataxia	periodontal fluid	NEG	NEG
DAM	Warmblood	24	chestnut	M	dental consult	saliva	NEG	NEG
EVI	Warmblood	17	chestnut	M	healthy	saliva	NEG	NEG
FIF	Warmblood	20	chestnut	G	healthy	saliva	NEG	NEG
FJA	Icelandic horse	29	buckskin	G	dental consult	saliva	NEG	NEG
GRY	Warmblood	27	chestnut	G	dental consult	saliva	NEG	NEG
GYÖ	Shagya-Arabian	17	grey	M	healthy	saliva	NEG	NEG
HOP	Warmblood	18	chestnut	G	dental consult	periodontal fluid	NEG	NEG
LOR	Welsh cob	19	dun	G	dental consult	periodontal fluid	NEG	NEG
LRT	Warmblood	24	bay	G	dental consult	saliva	NEG	NEG
LUZ	Shetland pony	30	grey	M	dental consult	saliva	NEG	NEG
MRK	Warmblood x Shagya-Arabian	20	bay	G	dental consult	saliva	NEG	NEG
MTZ	Shetland pony	19	grey	G	dental consult	saliva	NEG	NEG
MRO	Warmblood	19	bay	G	dental consult	saliva	NEG	NEG
PON	Trakehner	19	bay	G	dental consult	saliva	NEG	NEG
RAI	Warmblood	18	bay	G	healthy	saliva	NEG	NEG
SCA	German Riding pony	18	bay	M	dental consult	saliva	NEG	NEG
SCK	Warmblood	17	bay	G	dental consult	saliva	NEG	NEG
SPY	Appaloosa	20	spotted	G	dental consult	saliva	NEG	NEG
STS	Noriker horse	18	black	M	healthy	saliva	NEG	NEG
VLX	Warmblood	24	chestnut	G	dental consult	saliva	NEG	**2**
WEM	Warmblood	23	chestnut	G	dental consult	saliva	NEG	NEG

**OCULAR SWABS FROM APPARENTLY TUMOR-FREE HORSES (n = 30; from 30 horses)**								
BEN	Trotter	25	chestnut	G	training horse	ocular swab	NEG	**POS**
BIL	Warmblood	19	bay	G	training horse	ocular swab	NEG	NEG
BUS	Half-bred	14	bay	M	sinusitis	ocular swab	NEG	**POS**
CAR	Trotter	15	bay	M	training horse	ocular swab	NEG	**5**
CLO	Warmblood	7	bay	G	sinusitis	ocular swab	NEG	**5a**
DLY	Haflinger	14	bay	M	intoxication	ocular swab	NEG	**5**
DUD	Trotter	14	bay	G	training horse	ocular swab	NEG	**2**
ELM	Shetland pony	20	grey	M	training horse	ocular swab	NEG	NEG
GOR	Haflinger	12	sorrel	M	training horse	ocular swab	NEG	NEG
GRD	Warmblood	17	?	S	training horse	ocular swab	NEG	**5**
HAN	Warmblood	24	grey	G	training horse	ocular swab	NEG	**POS**
JEN	Trotter	37	black	M	training horse	ocular swab	NEG	**POS**
LUX	Haflinger	11	sorrel	M	training horse	ocular swab	NEG	NEG
MSH	Icelandic horse	10	chestnut	G	recurrent epithelial erosion left eye	ocular swab	NEG	NEG
MIN	Trotter	?	bay	M	gastro consult	ocular swab	NEG	NEG
MIS	Haflinger	2	sorrel	M	training horse	ocular swab	NEG	NEG
RAM	Warmblood	24	bay	G	training horse	ocular swab	NEG	**2**
ROS	Warmblood	37	chestnut	G	training horse	ocular swab	NEG	NEG
SBL	Haflinger	14	sorrel	M	training horse	ocular swab	NEG	NEG
SMY	Trotter	11	bay	G	lameness	ocular swab	NEG	NEG
SRG	Warmblood	14	chestnut	G	training horse	ocular swab	NEG	NEG
SJÖ	Icelandic horse	2	chestnut	S	castration	ocular swab	NEG	NEG
SNJ	Warmblood	27	bay	M	training horse	ocular swab	NEG	NEG
SON	Haflinger	10	sorrel	M	training horse	ocular swab	NEG	**2**
SPH	Shetland pony	16	grey	M	training horse	ocular swab	NEG	NEG
TPS	Shetland pony	7	grey	M	training horse	ocular swab	NEG	NEG
VLL	Warmblood	3	chestnut	G	dental consult	ocular swab	NEG	**5a**
VJE	Trotter	11	bay	G	training horse	ocular swab	NEG	**POS**
WGA	Haflinger	13	Sorrel	M	training horse	ocular swab	NEG	NEG
ZOL	Warmblood	12	chestnut	G	training horse	ocular swab	NEG	**2**
**VULVOVAGINAL SWABS FROM TUMOR-FREE MARES (n = 49, from 49 horses)**								
AP	Noriker horse	7	bay	M	lameness	vulvovaginal swab	NEG	**5a**
ANI	Noriker horse	15	spotted	M	dental consult	vulvovaginal swab	NEG	NEG
ANN	Shetland pony	10	chestnut	M	training horse	vulvovaginal swab	NEG	NEG

ARK	Warmblood	5	chestnut	M	insemination	vulvovaginal swab	NEG	**5a**
BBI	Shetland pony	1	chestnut	M	training horse	vulvovaginal swab	NEG	**2**
BCH	Shetland pony	19	skewbald	M	fracture	vulvovaginal swab	NEG	NEG
CMN	Trotter	11	bay	M	training horse	vulvovaginal swab	NEG	NEG
CHY	Knabstrupper	8	spotted	M	unrelated clinical study	vulvovaginal swab	NEG	NEG
CDL	Shetland pony	4	NI	M	insemination	vulvovaginal swab	NEG	NEG
CLD	Shetland pony	20	black	M	training horse	vulvovaginal swab	NEG	NEG
DOR	Shetland pony		bay	M	training horse	vulvovaginal swab	NEG	NEG
ERB	Shetland pony	11	grey	M	training horse	vulvovaginal swab	NEG	**2**
EVI	Hanoverian	17	chestnut	M	trauma	vulvovaginal swab	NEG	NEG
FIN	Icelandic horse	7	black	M	colic	vulvovaginal swab	NEG	NEG
FOR	Trotter x Arabian	12	bay	M	unrelated clinical study	vulvovaginal swab	NEG	**2** **5a**
FRZ	Haflinger x	7	chestnut	M	training	vulvovaginal swab	NEG	NEG
GIS	Haflinger	4	sorrel	M	training horse	vulvovaginal swab	NEG	NEG
GOR	Haflinger	8	sorrel	M	training horse	vulvovaginal swab	NEG	NEG
GYÖ	Shagya-Arabian	17	grey	M	unrelated clinical study	vulvovaginal swab	NEG	NEG
IND	Trotter	10	bay	M	training horse	vulvovaginal swab	NEG	NEG
JEN	Trotter	37	black	M	training horse	vulvovaginal swab	NEG	**2**
KRI	Trotter	13	bay	M	training horse	vulvovaginal swab	NEG	NEG
LYM	Warmblood	7	grey	M	neck trauma	vulvovaginal swab	NEG	NEG
LAL	Warmblood	13	NI	M	dietary consult	vulvovaginal swab	NEG	NEG
LES	Warmblood	5	chestnut	M	unrelated clinical study	vulvovaginal swab	NEG	NEG
LUN	Lipizzaner	8	grey	M	training horse	vulvovaginal swab	NEG	NEG
MII	Pony	12	spotted	M	unrelated clinical study	vulvovaginal swab	NEG	**5a**
MNN	Haflinger	11	sorrel	M	pododermatitis	vulvovaginal swab	NEG	NEG
PLA	Warmblood	12	bay	M	sarcoid patient, scurf	vulvovaginal swab	NEG	NEG
PRC	Warmblood	23	chestnut	M	ophthalmological consult	vulvovaginal swab	NEG	NEG
RIB	Warmblood	5	bay	M	trauma	vulvovaginal swab	NEG	NEG
SAB	Warmblood	28	bay	M	training horse	vulvovaginal swab	NEG	NEG
SAL	Shetland pony	15	chestnut	M	training horse	vulvovaginal swab	NEG	NEG
SMB	Haflinger	4	sorrel	M	immunological castration	vulvovaginal swab	NEG	NEG
SPP	Connemara	16	grey	M	oral SCC	vulvovaginal swab	NEG	NEG
SIN	Warmblood	19	dark bay	M	training horse	vulvovaginal swab	NEG	NEG
SON	Haflinger	10	sorrel	M	training horse	vulvovaginal swab	NEG	NEG
SNJ	Warmblood	23	bay	M	training horse	vulvovaginal swab	NEG	**5a**
SPH	Shetland pony	12	grey	M	insemination	vulvovaginal swab	NEG	NEG
STS	Noriker horse	19	black	M	training horse	vulvovaginal swab	NEG	NEG

STF	Shetland pony	12	grey	M	training horse	vulvovaginal swab	NEG	NEG
SUS	Haflinger	9	sorrel	M	training horse	vulvovaginal swab	NEG	**5a**
TNJ	Shetland pony	18	black	M	training horse	vulvovaginal swab	NEG	NEG
TIZ	Shetland pony	8	chestnut	M	training horse	vulvovaginal swab	NEG	**2**
TOR	Haflinger	3	sorrel	M	training horse	vulvovaginal swab	NEG	**5**
VLT	Trotter	29	bay	M	trauma, dental consult	vulvovaginal swab	NEG	NEG
WHI	Warmblood	8	chestnut	M	coughing	vulvovaginal swab	NEG	NEG
WLG	Haflinger	13	Sorrel	M	training horse	vulvovaginal swab	NEG	NEG
WDF	Warmblood	7	bay	M	insemination	vulvovaginal swab	NEG	**5a**
**URETHRAL FOSSA SWABS AND SMEGMA FROM TUMOR-FREE MALE HORSES (n = 34: from 34 horses)**								
ALE	Trakehner	18	chestnut	G	dental consult	smegma	**2**	NEG
BAC	Warmblood	12	skewbald	G	dental consult	smegma	NEG	**5a**
COL	Warmblood	3	black	G	pharyngeal constipation	smegma	NEG	NEG
CVO	Lipizzaner	2	grey	S	grass thickness	smegma	NEG	**5a**
DAT	Warmblood	10	chestnut	G	endoscopy	smegma	NEG	**5a**
DIDI	Shetland pony	4	grey	S	training horse	urethral fossa swab	NEG	**2**
DDO	Shetland pony	3	grey	G	birth	smegma	NEG	NEG
DUF	Pony	4	grey	S	training horse	urethral fossa swab	NEG	**5**
DUS	Shetland pony	3	grey	G	training horse	urethral fossa swab	NEG	**2**
EDG	Shetland pony	18	black	S	training horse	smegma	NEG	NEG
FER	Shetland pony	12	piebald	S	training horse	smegma	NEG	NEG
FND	Warmblood	8	chestnut	G	unrelated clinical study	urethral fossa swab	NEG	NEG
FOA	Warmblood	1	bay	S	umbilical hernia	smegma	NEG	**2**
FRZ	Shetland pony	18	black	S	training horse	smegma	NEG	NEG
GAU	Warmblood	21	grey	G	dental consult	smegma	NEG	NEG
GRW	Warmblood	16	bay	S	training horse	smegma	NEG	NEG
HNY	German Riding pony	16	chestnut	G	unrelated clinical study	urethral fossa swab	NEG	NEG
JEP	Hucul	10	black	G	pulmo-consult	smegma	NEG	**POS**
JOC	Shetland pony	18	grey	S	training horse	urethral fossa swab	NEG	NEG
KLB	Warmblood	5	grey	G	melanoma	smegma	NEG	NEG
LCL	Thoroughbred	1	chestnut	S	umbilical hernia	smegma	NEG	2
MER	Pony x Shagya Arabian	5	grey	G	unrelated clinical study	urethral fossa swab	NEG	**2**
MIC	Shetland pony	2	dark grey	S	Training horse	smegma	NEG	NEG
MRW	Shetland pony	4	grey	G	training horse	smegma	NEG	**2**
MUF	Shetland pony	12	chestnut	S	training horse	smegma	NEG	NEG
PIC	Warmblood	9	chestnut	G	colic	smegma	NEG	NEG

SOL	Thoroughbred	3	NI	S	lethal sinusitis	smegma	NEG	**5**
SVÖ	Icelandic horse	4	black	S	castration	smegma	NEG	**5a**
TIL	Shetland pony	4	grey	S	training horse	urethral fossa swab	NEG	NEG
TOM	Shetland pony	2	grey	G	training horse	smegma	NEG	**2**
TRI	Shetland pony	1	NI	S	training horse	urethral fossa swab	NEG	**2**
TWI	Shetland pony	17	chestnut	S	training horse	smegma	NEG	**2**
WKA	Warmblood	16	chestnut	S	training horse	smegma	NEG	NEG
WDS	Warmblood	19	black	G	colic	smegma	NEG	NEG

EcPV: 2 = EcPV2, 3 = EcPV3; 4 = EcPV4; 5 = EcPV5; EHV type: equid Herpesvirus type; 2 = EHV2; 3 = EHV3; 5 = EHV5; 5a = AsHV5; NEG: negative: POS: Herpesvirus PCR positive, but herpesvirus type not identified; M: mare; G: gelding; S: stallion
